# The effect of cigarette smoking on the oral and nasal microbiota

**DOI:** 10.1186/s40168-016-0226-6

**Published:** 2017-01-17

**Authors:** Guoqin Yu, Stephen Phillips, Mitchell H. Gail, James J. Goedert, Michael S. Humphrys, Jacques Ravel, Yanfang Ren, Neil E. Caporaso

**Affiliations:** 1 Genetic Epidemiology Branch, Division of Cancer Epidemiology and Genetics, National Cancer Institute, NIH, DHHS, 9609 Medical Center Drive, Room 6E508, Bethesda, MD 20892-9769 USA; 2 Eastman Institute of Oral Health, University of Rochester, Rochester, NY USA; 3 Biostatistics Branch, Division of Cancer Epidemiology and Genetics, National Cancer Institute, NIH, DHHS, Bethesda, MD USA; 4 Infections and Immunoepidemiology Branch, Division of Cancer Epidemiology and Genetics, National Cancer Institute, NIH, DHHS, Bethesda, MD USA; 5 Institute for Genome Sciences, University of Maryland School of Medicine, Baltimore, MD USA

**Keywords:** Oral cavity, Nasal cavity, Microbiota, 16S rRNA and cigarette smoke

## Abstract

**Background:**

The goal of the study was to investigate whether cigarette smoking alters oral and nasal microbial diversity, composition, and structure. Twenty-three current smokers and 20 never smokers were recruited. From each subject, nine samples including supra and subgingiva plaque scrapes, saliva, swabs from five soft oral tissue sites, and one nasal swab from both the anterior nares were collected. 16S rRNA V3-V4 region was sequenced for microbial profiles.

**Results:**

We found that alpha diversity was lower in smokers than in nonsmokers in the buccal mucosa, but in other sample sites, microbial diversity and composition were not significantly different by smoking status. Microbial profiles differed significantly among eight oral sites.

**Conclusions:**

This study investigates the effect of cigarette smoking on different sites of the oral cavity and shows a potential effect of cigarette smoking on the buccal mucosa microbiota. The marked heterogeneity of the oral microbial ecosystem that we found may contribute to the stability of the oral microbiota in most sites when facing environmental perturbations such as that caused by cigarette smoking.

**Electronic supplementary material:**

The online version of this article (doi:10.1186/s40168-016-0226-6) contains supplementary material, which is available to authorized users.

## Background

**Fig. 1:**
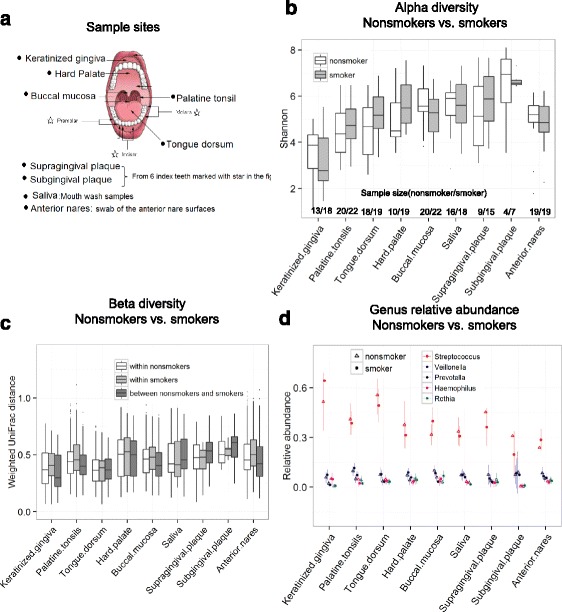
**a** Sampling of nine samples from each subject following the protocols used by HMP. **b** Shannon’s index by smoking status and sample sites. The Shannon index did not significantly differ by smoking status in any sample sites. **c** Within- and between-group (smoker, nonsmoker) weighted UniFrac distance (beta diversity). Within- versus between-group differences were not statistically significant by permutational multivariate analysis of variance (adonis). In Figure b and c, the *boxes* are interquartile range (IQR); median values are the *bands within the boxes*; the *lines outside the boxes* are 1.5-times IQR; *dots* are outliers. **d** Mean and 95% interval of genus-relative abundance. Streptococcus was most abundant in all sites, but no genus was significantly different in relative abundance by smoking status according to the Wilcoxon rank-sum test with Bonferroni correction. Only the five most abundant genera are shown

Cigarette smoke has adverse effects on human health. Smokers have increased risk of developing diseases such as lung and other cancers, chronic obstructive pulmonary disease, cardiovascular disease, and periodontitis [1]. The microbial communities in the mouth and the nose have direct contact with cigarette smoke and may thus be affected by it. Cigarette smoke contains numerous toxicants to which smokers are regularly exposed on a periodic basis. These toxicants can potentially perturb the microbial ecology of the mouth via antibiotic effects, oxygen deprivation, or other potential mechanisms [2]. The current study examined this question by characterizing the microbiota in eight oral sites and a nasal swab (Fig. 1a) and comparing them between never smokers and current smokers.

## Results

The characteristics of the study subjects were shown in Additional file 1: Table S1. No difference in age, gender, race, and alcohol drink were found by smoking status. Smokers had slightly higher periodontal screening and recording (PSR) scores than nonsmokers.

By the Wilcoxon rank-sum test, smokers did not differ significantly from nonsmokers at any site on any measure of alpha diversity, beta diversity, or taxa-relative abundance with one exception—PD_whole tree diversity was lower in the smokers’ buccal mucosa (*p* = 0.05) (Fig. 1b–d, Additional file 2: Table S2). Based on the *t* test for the buccal mucosa, smokers had marginally lower observed species (*p* = 0.046), PD_whole tree (*p* = 0.032), and nearly significant lower Shannon index (*p* = 0.074). Thus, it appears that smokers had lower alpha diversity than that in the nonsmokers’ buccal mucosa based on both Wilcoxon rank-sum and *t* tests. We plotted the mean difference between smokers and nonsmokers for observed species and PD_whole tree (Additional file 1: Figure S1). For most sites, we can exclude large differences in means, but for sub and supragingival plaques, the confidence intervals are wide, reflecting small sample size. Thus, we cannot exclude the possibility of a substantial smoking effect at these two sites. Moreover, Additional file 2: Figure S1 indicates that alpha diversity in the buccal mucosa is lower in smokers as described above.

**Fig. 2:**
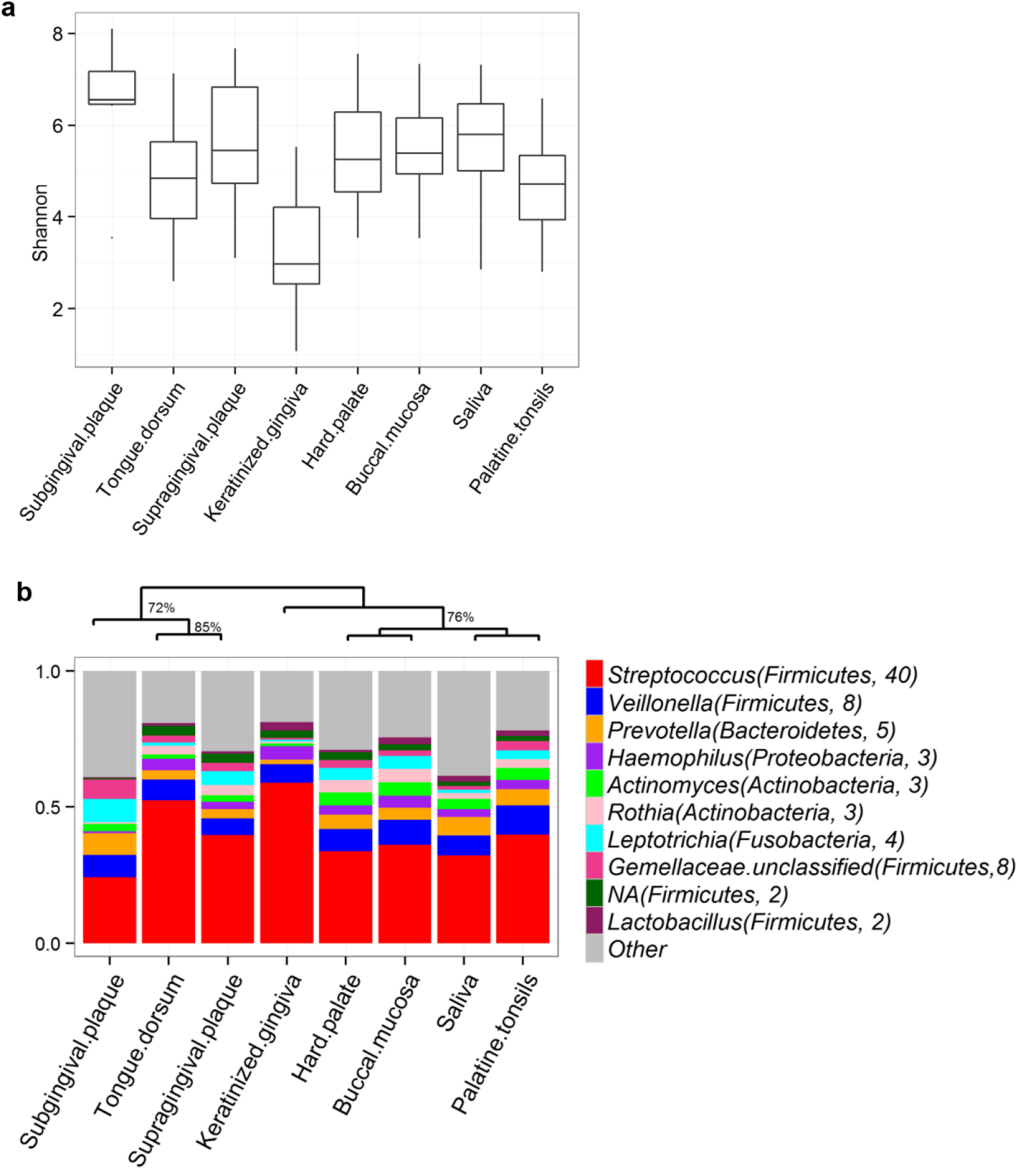
**a** Comparison of Shannon’s index (alpha diversity) across eight oral sites. The pairwise comparison by Wilcoxon signed-rank tests are shown in Additional file 2: Table S3. **b** Genus-level profiles and *dendrogram* showing similarity and difference among oral sites. The *dendrogram* at the top was based on hierarchical clustering using complete linkage of Bray-Curtis distance of the OTU table. Splits seen in at least 70% of 1000 bootstrap sampling are shown. Each *vertical bar* represents the profile averaged within each sample site. The average relative abundance (%) is shown in *parenthesis* after each genus. Only the most abundant genera are shown

Regardless of the smoking status, microbiota among the oral sites differed greatly (Fig. 2, Additional file 2: Table S3). We found no difference in any microbial measurement by age, gender, race, alcohol consumption, and PSR score (data not shown).

## Discussion

Our study reported an effect of cigarette smoke on the oral microbiota in the buccal mucosa. Although our sample size is modest, it is remarkable that overall, we found no associations between microbial features and smoking status in other oral sites. Our results for the buccal mucosa should be examined in other studies since we tested multiple sites. Previous studies have shown inconsistent results regarding the effect of smoking on the oral microbiota. For example, an altered microbiota composition was noted in studies of 62 swabs from the tonsillar pillars [3], 200 subgingival samples [4], 30 marginal and subgingival plaque and gingival crevicular fluid samples [5], and oral wash samples comparing 13–41 current smokers with 77–194 never smokers in 4 groups [6] and 64 saliva samples [7] by sequencing 16S rRNA gene and in a study of 292 stimulated oral samples by Human Oral Microbe Identification Microarray (HOMIM) [8]. These studies, however, reported that different taxa were changed by smoking status. Some studies [9–11] reported no association between oral microbiota and smoking. Heterogeneity in smoking definitions, oral sites sampled, study population, and inclusion/exclusion of participants with related conditions (i.e., periodontitis), and in methods for collecting the specimens, likely account for some of the inconsistency with respect to smoking. In addition, our results suggested that smoking did not affect the oral microbiota with a large effective size. Large studies capable of exploring smaller magnitude effects of smoking on specific oral niches are needed to fully understand subtle smoking-induced alteration in microbiota.

Data for smoking effects on the nasal microbiota are sparser and even less clear. In contrast to our null results, swabs from the left and right nasopharynx in 62 subjects found significant differences in relative abundance of diverse taxa by smoking status, but 55% of these smoking-associated taxa were found only in the left or right nasopharynx, suggesting inconsistency [3].

Differences of microbiota across oral sites, regardless of smoking, point to distinct microbial niches. Consistent with our findings and with the Human Microbiome Project (HMP) [12], a recent study of 66 Chinese subjects showed significant differences in microbiota composition among the buccal mucosa, saliva, and dental plaque sites [13]. Different oral sites harbor a distinct microbiota, suggesting that the various oral surfaces might provide different receptors for bacterial adhesion, species interactions, and environmental conditions (e.g., oxygen level) for microbial survival and growth.

Ecological studies have suggested that more diverse and complex communities are generally more resistant and resilient to perturbations [14]. In the HMP, the oral microbiota, despite its heterogeneity across subsites, was more stable over time than the other body site evaluated [12]. As shown here, the oral cavity is a highly heterogeneous ecological system containing distinct microbial niches. In addition, it is well accepted that microbial organisms living on oral surfaces can switch from a free-living state to a sessile mode in biofilms with advantageous properties that include enhanced tolerance to many adverse conditions including antimicrobial agents [15]. Consistent with this view, a recent study of 66 healthy subjects with saliva samples at baseline, immediately and 1, 2, 4, and 12 months after antibiotics usage found that saliva microbial composition remained stable [16]. Likewise, our findings suggest that the microbial communities in the oral cavity might be resistant or resilient to disturbances such as cigarette smoking.

Strengths of the current study include extensive sampling of the oral cavity with a well-developed protocol, careful matching of case and control groups, a contrast of never with heavy smokers, state-of-the-art assays including quality control samples for batch effects, and rigorous statistical analysis. These methods should have identified significant associations with smoking status in the most sampled sites. Weaknesses include the modest sample size, especially for subgingival plaque samples due to sequencing failure. The study only allows us to detect the effect of smoking with a large effect size on the oral microbiota. Subtle effects or temporal effects of smoking could not be examined in this study.

## Conclusions

Our study showed that cigarette smoking had a significant effect on the microbiota of the buccal mucosa, but not in other oral sites and nasal cavity. The oral cavity is heterogeneous with distinct communities across sites, which may contribute to its stability in the face of potential perturbing factors such as smoking. Larger studies are needed to further examine oral and nasal microbiota effects produced by behaviors such as cigarette smoking.

## Methods

### Study subjects

Following the approval by the institutional review boards of the National Cancer Institute and University of Rochester, 23 current smokers (median duration, 15 years; median intensity, 15 cigarettes per day) and 20 nonsmokers (<100 cigarettes in a lifetime) were recruited at Eastman Institute of Oral Health, University of Rochester. All the subjects signed informed consent and filled out questionnaires. Individuals with antibiotic usage or professional dental cleaning within the last 3 months or diagnosed with periodontal disease or cancer or losing >1 tooth were excluded. Participants were screened by PSR index estimated at the time of recruitment. Groups were frequency-matched for gender and race (Additional file 1: Table S1).

### Biospecimen collection

From each subject, we sought nine samples, including supra and subgingiva plaque, saliva, swabs from five soft tissue sites, and one nasal swab from both the anterior nares. Figure 1a shows the detailed sample collection locations in the oral cavity. The samples were collected by following the procedure of Human Microbiome Project (http://hmpdacc.org/doc/HMP_MOP_Version12_0_072910.pdf).

### 16S rRNA gene sequence analysis

The DNA was extracted from samples as described previously [17]. The V3-V4 regions of the 16S rRNA gene were amplified and sequenced on an Illumina MiSeq instrument using the 300 paired-end protocol at the Institute of Genome Sciences, Genomic Resource Center, University of Maryland School of Medicine [17]. The sequence data were submitted to NCBI BioProject (http://www.ncbi.nlm.nih.gov/bioproject) under accession number PRJNA316469.

Sequence reads were processed to remove low quality and short reads (see details in [18]). The remaining reads (18,497 ± 14,130 reads/sample) were clustered into Operational Taxonomy Units (OTUs) at 97% identity using the command pick_open_reference_otus.py and Greengenes database as the reference (version 13_8) [19] in Quantitative Insights into Microbial Ecology (QIIME 1.8.0) [20]. The default parameters were used except the method of usearch61 and percent_subsample of 0.1. OTUs with only one read were excluded.

Alpha diversity was estimated as the number of OTUs (Observed_species), Shannon’s Index (using information of OTU frequency) [21], and phylogenetic diversity (using information of phylogenetic relationship of different OTUs) (PD_whole_tree) [22]) by averaging over 20 rarefied tables (1000 reads/sample). Taxonomic beta diversity was measured as unweighted and weighted UniFrac distance based on the OTU table [23]. Relative abundance of taxa was calculated from unrarefied OTU table. To rule out batch effects, 19 random samples were selected to examine the difference within and between batches, and no difference was found in alpha and beta diversity measures (Additional file 1: Figure S2).

### Statistical analysis

The Wilcoxon rank-sum test was used to examine gastric microbiota alpha diversity and taxa-relative abundance differences between groups. For the buccal mucosa, 95% confidence intervals for differences in mean alpha diversity were based on t-statistics. The Spearman correlation was used to examine the correlation between continuous variables. Bonferroni correction was used to adjust for tests of multiple taxa. Permutational multivariate analysis of variance (PERMANOVA, adonis) was used to examine the association between unweighted/weighted UniFrac distance and smoking status/other demographic variables. *P* values less than 0.05 were considered significant after adjustment for multiple tests.

## Additional files

Additional file 1: Table S1. Summary of study subjects by smoking status. **Figure S1.** Mean difference between smokers and nonsmokers in observed species (a) and PD_whole_tree (b) with 95% confidence interval based on t-distribution. The black dots are mean difference between smokers and nonsmokers (smokers, nonsmokers). **Figure S2.** Box plot showing no difference of within- and between-plate/batch variation in alpha diversity (Shannon) and beta diversity (unweighted and weighted UniFrac). Boxes are interquartile range (IQR), median values are bands within the boxes, lines outside the boxes are 1.5-times IQR, and dots are outliers. (DOC 391 kb)
Additional file 2: Table S2. Comparison by smoking status in alpha diversity and relative abundance of top ten abundant genera. **Table S3.** *P* values based on the Wilcoxon signed-rank test for the Shannon index differences among eight oral sites. (XLS 50 kb)

## Abbreviations

HMP: Human microbiome project
OTU: Operational taxonomical unit
PD_whole_tree: Phylogenetic diversity
PERMANOVA: Permutational multivariate analysis of variance
PSR: Periodontal screening and recording
QIIME: Quantitative insights into microbial ecology
