# Supplementary material for: The effect of cigarette smoking on the oral and nasal microbiota
Source: Microbiome. 2017 Jan 17;5:3. doi: 10.1186/s40168-016-0226-6 (PMC5240432; doi:10.1186/s40168-016-0226-6)
Supplement: Additional file 1: Table S1. — Summary of study subjects by smoking status. Figure S1. Mean difference between smokers and nonsmokers in observed species (a) and PD_whole_tree (b) with 95% confidence interval based on t-distribution. The black dots are mean difference between smokers and nonsmokers (smokers, nonsmokers). Figure S2. Box plot showing no difference of within- and between-plate/batch variation in alpha diversity (Shannon) and beta diversity (unweighted and weighted UniFrac). Boxes are interquartile range (IQR), median values are bands within the boxes, lines outside the boxes are 1.5-times IQR, and dots are outliers. (DOC 391 kb) [file 40168_2016_226_MOESM1_ESM.doc]

**Table S1, Summary of study subjects by smoking status**

|  | Nonsmoker (n=20) | Smoker (n=23) | P value |
| --- | --- | --- | --- |
| Age (years) | 34(28-50) | 45(33-49) | 0.55 |
| Gender (M/F) | 10/10 | 13/10 | 0.77 |
| Race (black/white) | 9/11 | 11/12 | 1 |
| Cigarettes per day (number) | NA | 15(10-20) | NA |
| Total years smoking (years) | NA | 15(11-29) | NA |
| Alcohol drink (rarely/weekly) | 17/3 | 20/3 | 1 |
| PSR score | 4.5 (4.0 -6.0) | 6.0 (5.0 -8.0) | 0.03 |

Note: Note: Median(interquartile range) or counts are shown. P values were based on Wilcoxon test for continuous variables, Fisher's exact test for counts.


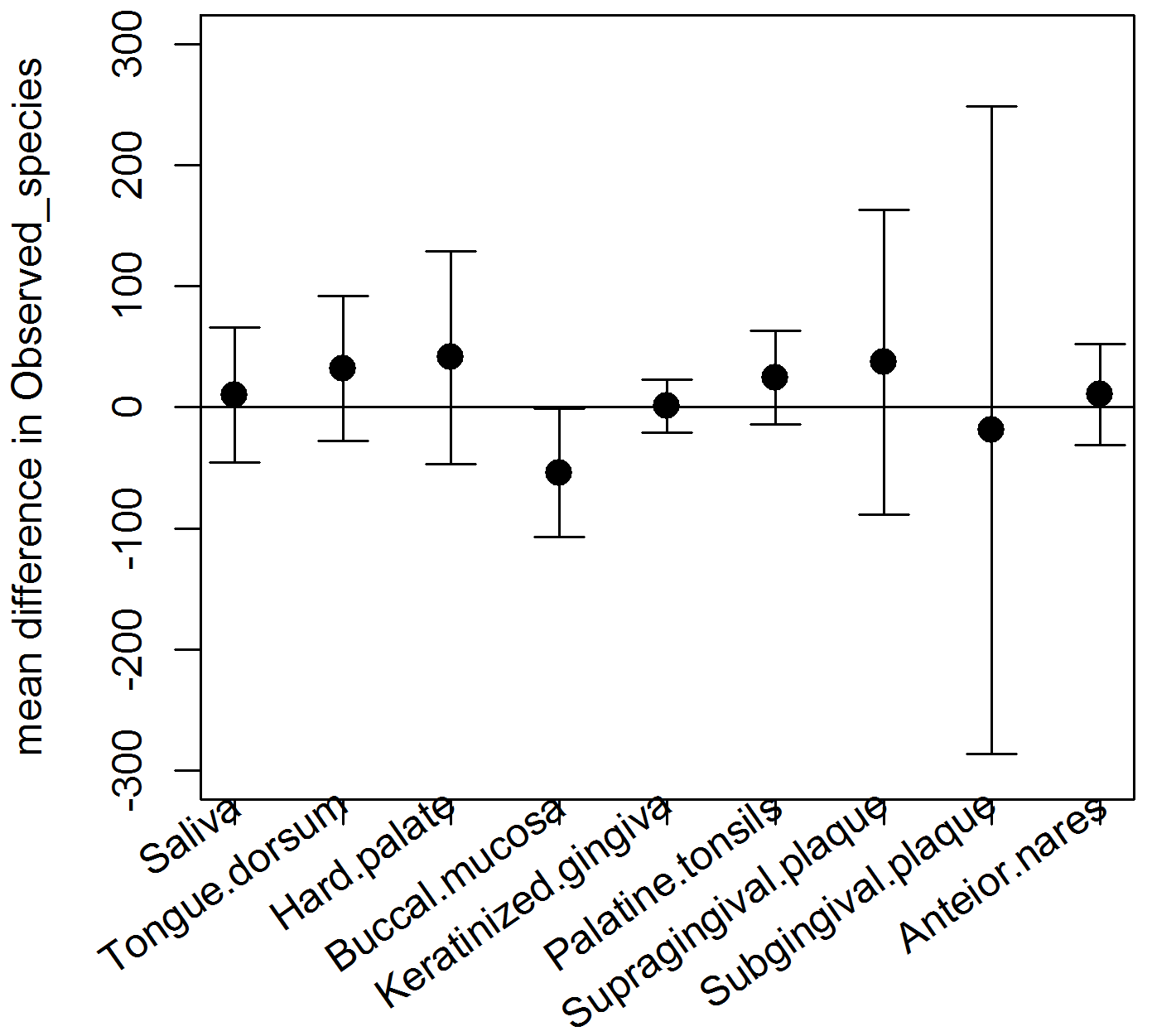

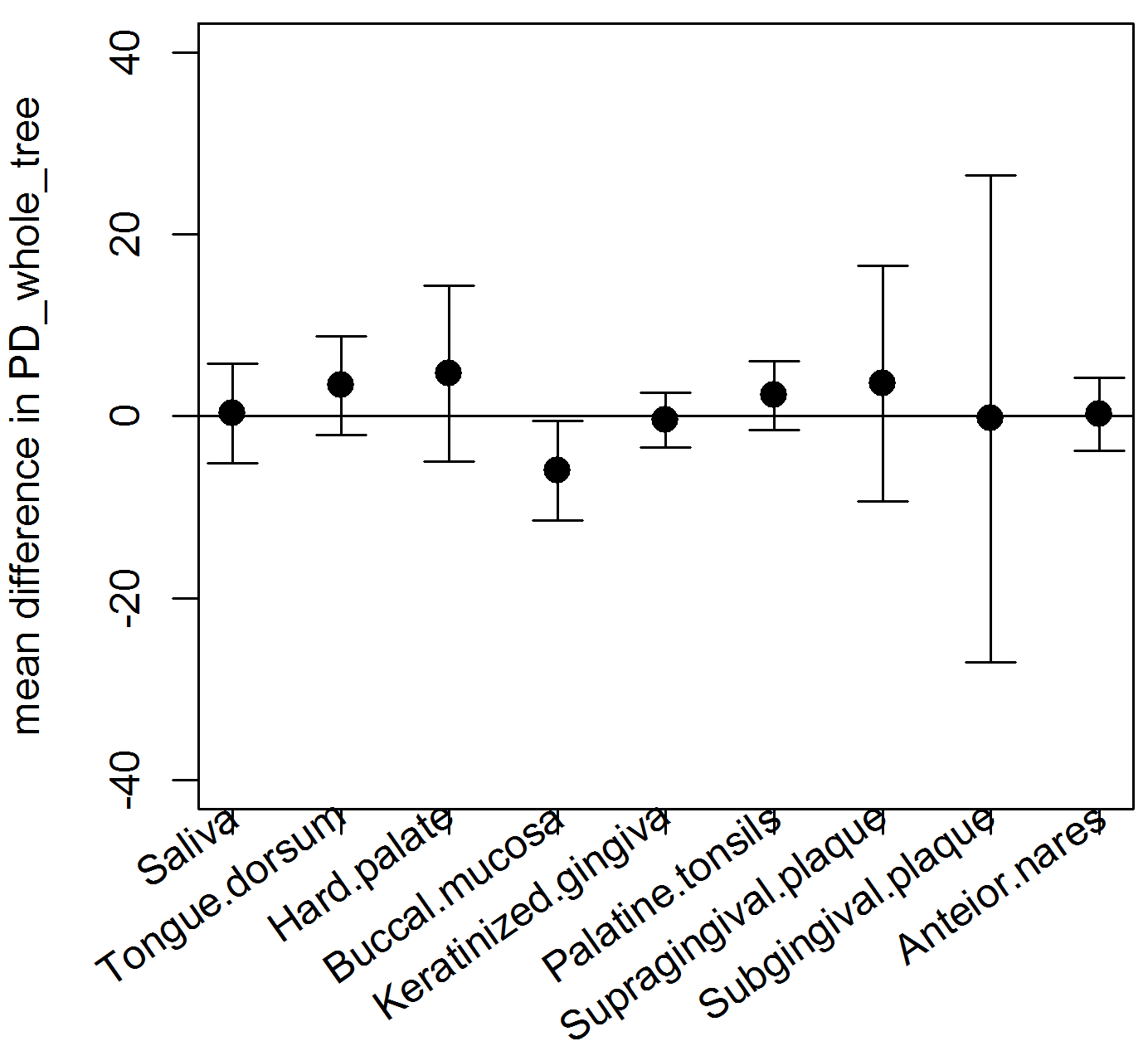


**a**

**b**

**Figure S1,** Mean difference between smoker and nonsmokers in observed species (a) and PD_whole_tree (b) with 95% confidence interval based on t-distribution. The black dots are mean difference between smokers and nonsmokers (smokers - nonsmokers).


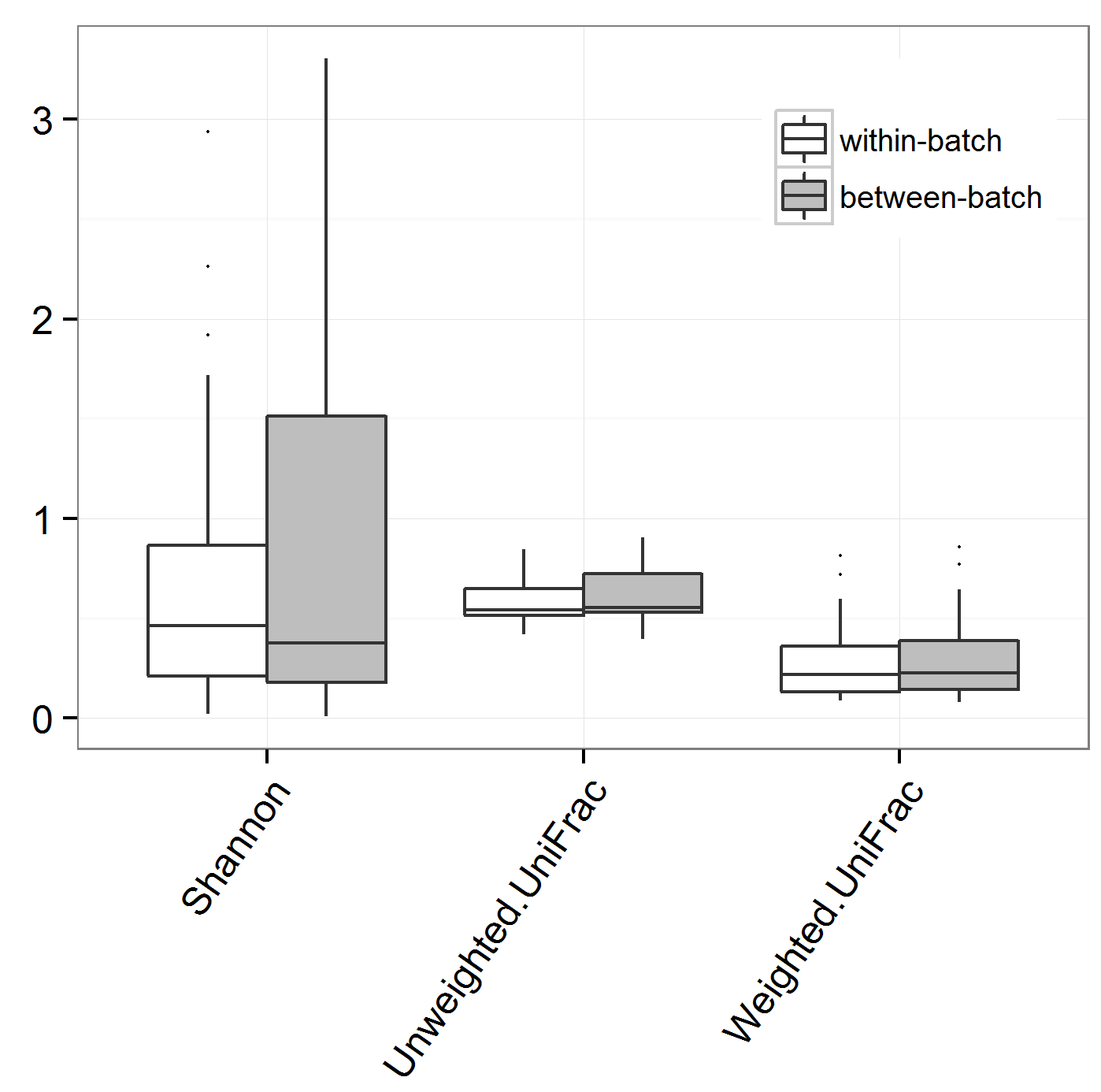


**Figure S2**, Box plot showing no difference of within- and between-plate/batch variation in alpha diversity (Shannon) and beta diversity (unweighted and weighted UniFrac). Boxes are interquartile range (IQR); median values are bands within the boxes; lines outside the boxes are 1.5-times IQR; dots are outliers.
